# A variational Bayes algorithm for fast and accurate multiple locus genome-wide association analysis

**DOI:** 10.1186/1471-2105-11-58

**Published:** 2010-01-27

**Authors:** Benjamin A Logsdon, Gabriel E Hoffman, Jason G Mezey

**Affiliations:** 1 Department of Biological Statistics and Computational Biology, Cornell University, Ithaca, NY, USA; 2 Department of Genetic Medicine, Weill Cornell Medical College, NY, NY, USA

## Abstract

**Background:**

The success achieved by genome-wide association (GWA) studies in the identification of candidate loci for complex diseases has been accompanied by an inability to explain the bulk of heritability. Here, we describe the algorithm V-Bay, a variational Bayes algorithm for multiple locus GWA analysis, which is designed to identify weaker associations that may contribute to this missing heritability.

**Results:**

V-Bay provides a novel solution to the computational scaling constraints of most multiple locus methods and can complete a simultaneous analysis of a million genetic markers in a few hours, when using a desktop. Using a range of simulated genetic and GWA experimental scenarios, we demonstrate that V-Bay is highly accurate, and reliably identifies associations that are too weak to be discovered by single-marker testing approaches. V-Bay can also outperform a multiple locus analysis method based on the lasso, which has similar scaling properties for large numbers of genetic markers. For demonstration purposes, we also use V-Bay to confirm associations with gene expression in cell lines derived from the Phase II individuals of HapMap.

**Conclusions:**

V-Bay is a versatile, fast, and accurate multiple locus GWA analysis tool for the practitioner interested in identifying weaker associations without high false positive rates.

## Background

Genome-wide association (GWA) studies have identified genetic loci associated with complex diseases and other aspects of human physiology [1,2]. All replicable associations identified to date have been discovered using GWA analysis techniques that analyze one genetic marker at a time [3]. While successful, it is well appreciated that single-marker analysis strategies may not be the most powerful approaches for GWA analysis [4]. Multiple locus inference is an alternative to single-marker GWA analysis that can have greater power to identify weaker associations, which can arise due to small allelic effects, low minor allele frequencies (MAF), and weak correlations with genotyped markers [4]. By correctly accounting for the effects of multiple loci, such approaches can reduce the estimate of the error variance, which in turn increases the power to detect weaker associations for a fixed sample size. Since loci with weaker associations may contribute to a portion of the so-called 'missing' or 'dark' heritability [5-7], multiple locus analyses have the potential to provide a more complete picture of heritable variation.

Methods for multiple locus GWA analysis must address a number of problems, including 'over-fitting' where too many associations are included in the genetic model, as well as difficulties associated with model inference when the number of genetic markers is far larger than the sample size [8]. Two general approaches have been suggested to address these challenges: hierarchical models and partitioning/classification. Hierarchical modeling approaches [9-14] employ an underlying regression framework to model multiple marker-phenotype associations and use the hierarchical model structure to implement penalized likelihood [10], shrinkage estimation [15], or related approaches to control over-fitting. These methods have appealing statistical properties for GWA analysis when both the sample size and the number of true associations expected are far less than the number of markers analyzed, which is generally considered a reasonable assumption in GWA studies [8]. Alternatively, partitioning methods do not (necessarily) assume a specific form of the marker-phenotype relationships but rather assume that markers fall into non-overlapping classes, which specify phenotype association or no phenotype association [13,16]. Control of model over-fitting in high dimensional GWA marker space can then be achieved by appropriate priors on marker representation in these classes [13].

Despite the appealing theoretical properties of multiple locus methods that make use of hierarchical models or partitioning, these methods have not seen wide acceptance for GWA analysis. There are at least two reasons for this. First, an ideal multiple locus analysis involves simultaneous assessment of all markers in a study and, given the scale of typical GWA experiments, most techniques are not computationally practical options [9,10,16-18]. Second, there are concerns about the accuracy and performance of multiple locus GWA analysis. This is largely an empirical question that needs to be addressed with simulations and analysis of real data.

Here we introduce the algorithm V-Bay, a (V)ariational method for (Bay)esian hierarchical regression, that can address some of the computational limitations shared by many multiple locus methods [9,10,16-18]. The variational Bayes algorithm of V-Bay is part of a broad class of approximate inference methods, which have been successfully applied to develop scalable algorithms for complex statistical problems, in the fields of machine learning and computational statistics [19-22]. The specific type of variational method implemented in V-Bay is a mean-field approximation, where a high dimensional joint distribution of many variables (in this case genetic marker effects) is approximated by a product of many lower dimensional distributions [23]. This method is extremely versatile and can be easily extended to a range of models proposed for multiple locus analysis [4,11,14,24].

The specific model implemented in V-Bay is a hierarchical linear model, which includes marker class partitioning control of model over-fitting. This is particularly well suited for maintaining a low false-positive rate when identifying weaker associations [13]. V-Bay implements a simultaneous analysis of all markers in a GWA study and, since the computational time complexity per iteration of V-Bay is linear with respect to sample size and marker number, the algorithm has fast convergence. For example, simultaneous analysis of a million markers, genotyped in more than a thousand individuals, can be completed using a standard desktop (with large memory capacity) in a matter of hours.

We take advantage of the computational speed of V-Bay to perform a simulation study of performance, for GWA data ranging from a hundred thousand to more than a million markers. In the **Results **we focus on the simulation results for single population simulations, but we also implement a version of the algorithm to accommodate known population structure and missing genotype data. We demonstrate that in practice, V-Bay consistently and reliably identifies both strong marker associations, as well as those too weak to be identified by single-marker analysis. We also demonstrate that V-Bay can outperform a recently proposed multiple locus methods that uses the least absolute shrinkage and selection operator (lasso) penalty [14], a theoretically well founded and widely accepted method for high dimensional model selection. V-Bay therefore provides a powerful complement to single-marker analysis for discovering weaker associations that may be responsible for a portion of missing heritability.

## Results and Discussion

### The V-Bay Algorithm

The V-Bay algorithm consists of two components: a hierarchical regression model with marker class partitioning and a variational algorithm for approximate Bayesian inference. The underlying hierarchical model of V-Bay is a Bayesian mixture prior regression [25] that has been previously applied to association and mapping problems [13]. The regression portion of this hierarchical model is a standard regression used to model genetic marker-phenotype associations, and allows for natural incorporation of population structure and other covariates. The model partitioning incorporates global features of genetic marker associations, which are assumed to be distributed among positive, negative, and zero effect classes. The zero effect class is used to provide a parametric representation of the assumption that most markers in GWA studies will not be linked to causative alleles and therefore do not have true associations with phenotype [13].

Approximate Bayesian inference with V-Bay is accomplished by an algorithm adapted from variational Bayes methods [26]. As with other variational Bayes methods, the goal of V-Bay is to approximate the joint posterior density of the hierarchical regression model with a factorized form and then to minimize the Kullback-Liebler (KL) divergence between the factorized form and the full posterior distribution [27]. This is accomplished by taking the expectation of the log joint posterior density, with respect to each parameter's density from the factorized form, and iterating until convergence [23]. The overall performance of V-Bay will depend on how well the factorized form approximates an informative mode of the posterior distribution of the hierarchical model. We have chosen a factorization with respect to each regression and hierarchical parameter, which appears to perform extremely well for identifying weak associations when analyzing simulated GWA data that include large numbers of genetic markers.

### Computational speed

The computational efficiency of V-Bay derives from two properties: it is a deterministic algorithm and the objective function has a factorized form. Since V-Bay is deterministic it does not need the long runs of Markov chains required by exact Bayesian MCMC algorithms [28]. For GWA analysis, these latter stochastic algorithms can be very slow to converge, particularly when marker numbers are large and when there are complex marker correlations produced by linkage disequilibrium [8]. The factorized form of V-Bay means that the minimization is performed with respect to each parameter independently, where each iterative update satisfies consistency conditions for maximizing the lower bound, given the state of the other parameters. Unlike univariate update algorithms, which may not necessarily have efficient updates with respect to the likelihood gradient function [4], the consistency conditions produced by the factorized form ensure that the univariate updates produce a computationally efficient approach to a KL-divergence minimum.

More precisely, V-Bay has linear time complexity scaling with respect to both marker number and sample size per iteration (Additional file 1, **Methods**). V-Bay therefore has better computational scaling properties than most currently proposed multiple locus algorithms for full likelihood or exact MCMC Bayesian analysis, when simultaneously considering all markers in a GWA study [9,10,16-18]. While the total time to convergence will depend on the true underlying genetic model, total computational times appear to be very tractable. As an example, using a dual-quad core Xeon 2.8 Ghz, with 16 Gb of memory, V-Bay converges in less than four hours for data sets in the range of 1 million markers, for a sample size of 200, and has average convergence around ten hours for sample sizes of 1000.

### Significance thresholds

**Figure 1:**
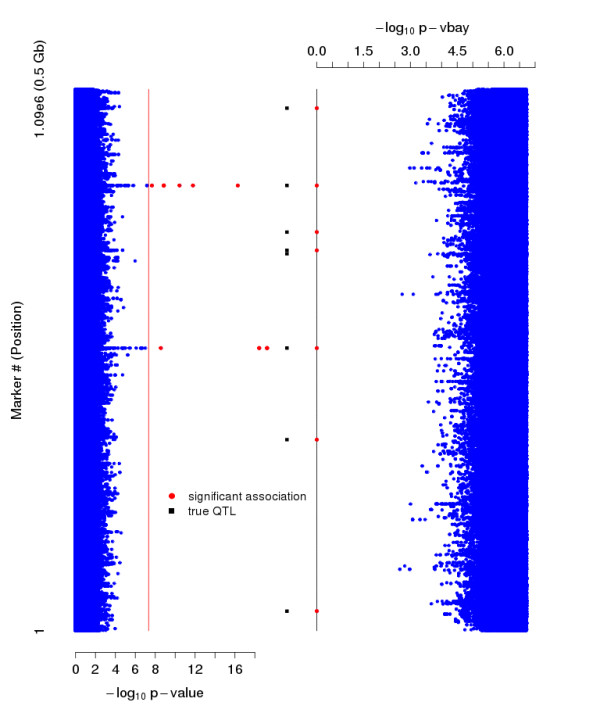
**Manhattan plots of the results of a single-marker (left) and V-Bay analysis (right) of a simulated GWA data set**. Data were simulated with a sample size of 200, one million markers, 8 loci with phenotype associations, and a total phenotype heritability of 0.9. The locations of the loci with phenotype associations are represented by the black squares. Each dot reflects the -log_10 _p-value resulting from single-marker analysis (left) and the -log_10 _p-vbay output of V-Bay (right), where non-significant associations are represented as blue dots. The markers above the red line for the single-marker analysis are significant when using a Bonferroni correction. The markers in red for the V-Bay analysis (connected by a black line) are significant using a conservative control of the false positive rate equal to a Bonferroni correction. In this case, the single-marker analysis correctly identifies two of the true associations, while V-Bay identifies 7 of the 8 true associations. This result was typical for our simulation analyses.

We assessed significance of marker associations using -log_10 _p-vbay, the negative log posterior probability of a marker being in either the positive or negative effect class. This is a natural statistic for deciding significance, since p-vbay is the (approximate posterior) probability that the marker has an association with the phenotype. While different significance thresholds based on -log_10 _p-vbay can be assigned to control false positive rate, as illustrated in Figure 1, the distribution of this statistic has an appealing property. The statistic has a value of zero for most of the true hits and there is a large gap (about 1-2 orders of magnitude) between significant markers and those with less significant scores. This is true even when the individual heritabilities of the true hits are low. This property of V-Bay is remarkably robust. A GWA practitioner using V-Bay can therefore easily identify a significant association (a 'hit') in practice when applying a conservative significance threshold.

### Performance of V-Bay compared to single-marker analysis

We empirically analyzed V-Bay performance on 150 simulated GWA data sets. Marker numbers for these data were one-hundred thousand, six-hundred thousand, or one million markers and were simulated using the approximate coalescent simulator MaCS [29]. We simulated a continuous phenotype with normally distributed error under the conditions listed in Table 1, where each GWA data set analyzed was produced by choosing a combination of these conditions. For these simulated data sets, we analyzed the performance of V-Bay compared to a single-marker analysis that was implemented by applying a linear regression model individually to each marker.

**Table 1:** Components and range of values used to simulate GWA data.

Component	Values
sample	200 or 1000
markers	0.1 to 1.0 million
missing	0% or 2%
loci	4, 8, or 32
effects	gamma(2,1) or fixed
heritability	0.5 or 0.9
populations	one or four

As illustrated in Table 2, V-Bay can perform better than single-marker analysis given a sufficient sample size or a sufficient number of loci with high individual heritabilities. Both the number of true associations identified and the amount of heritable variation explained can be greater when employing highly conservative false positive tolerances. For example, when using a false positive rate approaching a Bonferroni correction, V-Bay can on average double the number of associations found by single-marker analysis and can explain 20% more of the variance in phenotype under the most favorable conditions simulated. The reason for this increase in performance is that V-Bay has greater power to detect weaker (true) associations by accounting for the effects of multiple loci.

**Table 2:** Comparison of V-Bay and single-marker GWA analysis of simulated data for 1 million markers.

sample	loci	**(min/max)**^***a***^		**V-Bay min**			**single-marker min**	
200	4	0.24 (0.0032/0.75)	0.83	0.026	98.9	0.55	0.16	87.4
200	32	0.028 (6.7e-5/0.28)	0.053	0.033	26.9	0.072	0.050	35.3
1000	4	0.23 (0.0050/0.65)	1.00	0.0050	100	0.78	0.045	98.7
1000	32	0.028 (8.3e-5/0.30)	0.61	0.0037	95.6	0.32	0.0099	78.2

Phenotypes were simulated with a fixed total heritability of 0.9. The false positive rate was controlled to be < 10^-7 ^for both the V-Bay analysis and the single-marker analysis. (: average true positive rate)

^*a*^Average, maximum, and minimum individual heritabilities of the individual loci.

^*b*^The smallest individual heritability identified among the true positives.

^*c*^The average total heritability accounted for by the true positives identified.

**Figure 2:**
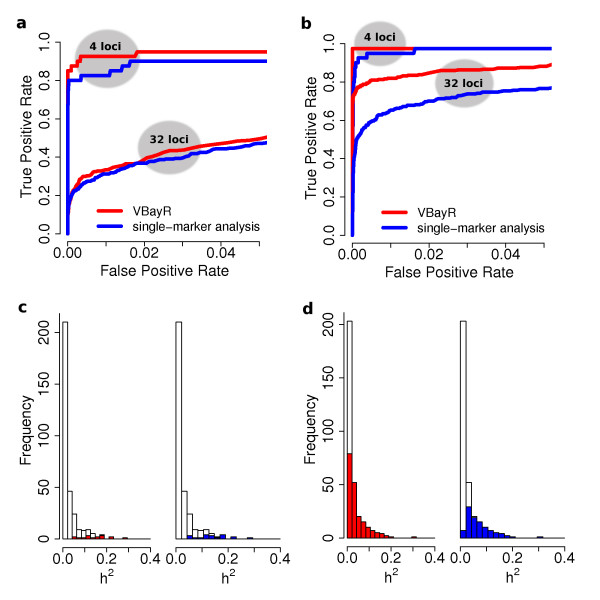
**Comparison of V-Bay and single-marker analysis for simulated GWA data**. The total heritability for the phenotype in each data set was controlled to be 0.9. The Receiver Operator Characteristic (ROC) curves in the upper graphs reflect the average across 10 replicate data sets that included (**a**) 200 samples and (**b**) 1000 samples. The lower graphs plot the distribution of individual heritabilities for the 32 loci simulations for the data sets that included (**c**) 200 samples and (**d**) 1000 samples, where the proportion of correctly identified loci for V-Bay are plotted in red and for single-marker analysis in blue when controlling the false positive rate at < 10^-7^.

Whether small associations are identified by V-Bay depends on the interplay between the sample size of the GWA study and the percentage of variation explained by the individual marker associations. For example, Figure 2a and 2b present the Receiver Operator Characteristic (ROC) curves comparing the performance of V-Bay and single-marker analyses for 10 replicate simulations, with 4 or 32 loci affecting a phenotype, total heritability of 0.9, and sample sizes of 200 or 1000, respectively (note that we use these high heritability cases for exploratory purposes; we also consider a total heritability of 0.5 in other simulations). With a sample size of 200 (Figure 2a), V-Bay outperforms single-marker analysis for the 4 loci simulations, and is about the same for the 32 loci simulations. The reason for the relative decrease in performance of V-Bay in this latter case is the average individual heritability associated with each associated marker is lower. Most of the true associations are therefore too small to detect even when controlling for the largest effects with a multiple locus method like V-Bay (Figure 2c). With a larger sample size however, V-Bay is able to detect a much larger proportion of the weaker associations in the case of 32 contributing loci (Figure 2d). Also, since there are more loci to detect with 32 loci, V-Bay has far better performance than single-marker analysis overall at a highly conservative false positive rate (< 10^-7^). Further simulations indicated that even for a uniform distribution of individual heritabilities (i.e. constant minor allele frequency and effect size), V-Bay performs better for similar sample sizes and individual heritabilities. For example, for 32 loci with a sample size of 1000, and false-discovery rate of 5.0% the average power of V-Bay was 93%. This is greater than the corresponding power of 72% for single-marker analysis with the same false-discovery rate. In general, regardless of sample size, if there are enough loci with associations that are not too weak, then V-Bay outperforms single-marker analysis.

V-Bay performance is a direct function of the individual heritabilities, and not the total heritability of the phenotype. The individual heritability is defined by both the minor allele frequency and the effect size (see **Methods**). Therefore loci with large effects may still have low individual heritabilities if the minor allele frequencies of the true loci are low (or vice versa). For example, for our simulations where the total heritability was controlled to be 0.5, and the individual heritabilities were shifted to be smaller overall, V-Bay performance was far closer to single-marker analysis. When we increased the individual heritabilities associated with associations in these simulations, while holding the total heritability at 0.5, V-Bay can outperform single-marker analysis. For all simulations, when an individual heritability falls below a certain threshold, neither approach could detect the association. There exists a limit to how weak an association can be and still be detected by V-Bay, given the sample size of the GWA study. Even in the worst case scenarios simulated, with many loci with small individual heritabilities and a small sample size, the performance of V-Bay was not significantly different from single-marker analysis across simulations. This result suggests that even if the number of loci were increased (i.e. the average individual heritability was decreased), the performance of V-Bay would at worst be the same as single-marker analysis.

**Figure 3:**
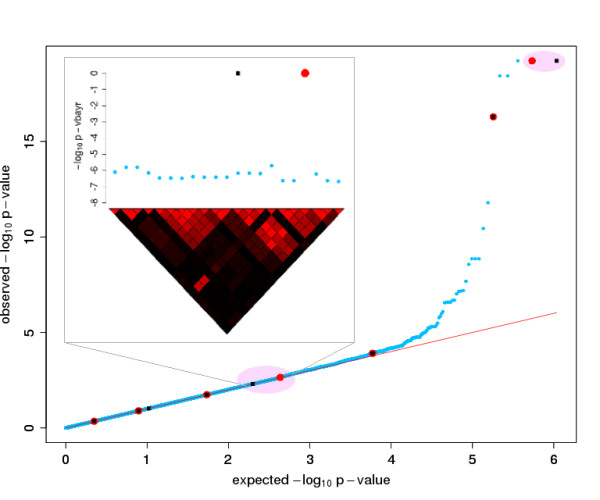
**Quantile-Quantile plot of the genome-wide p-values obtained in the single marker analysis of the data presented in Figure 1**. The seven associations correctly identified by V-Bay are circled in red. The locations of the loci with phenotype associations (black squares) and the results of the V-Bay analysis (red circles) are depicted with respect to their observed and expected quantiles from the single-marker analysis (blue circles). In this analysis, V-Bay is able to detect true associations that are undetectable with the single-marker analysis. The inset plot shows one of the hits from V-Bay that does not lie exactly on the marker in tightest linkage disequilibrium with the associated locus but is six SNPs away.

The inset in Figure 3 illustrates another appealing property of V-Bay. In contrast to a single-marker analysis, where each marker in a linkage disequilibrium block containing a true association will have an inflated -log_10 _p-value, V-Bay identifies only a single marker as significant, which is in high linkage disequilibrium with the true association. We found in our single population simulations that, while the specific marker assigned depends on the update order of the algorithm, the correlation between the marker and the causative allele averages *r*^2 ^= 0.75, with 28% of hits on markers in perfect linkage disequilibrium, and 52% of markers with *r*^2 ^≥ 0.9. V-Bay can therefore provide high mapping resolution within a linkage disequilibrium block.

### Comparison to the Lasso

The V-Bay algorithm was compared to the lasso, one of the only other currently proposed multiple locus methods that make use of a hierarchical regression model and have similar scaling properties to V-Bay [14]. For comparison to V-Bay, we use a form that implements a lasso type penalty [30], based on the algorithm presented in Wu et al. [14], modified to allow continuous phenotypes.

**Figure 4:**
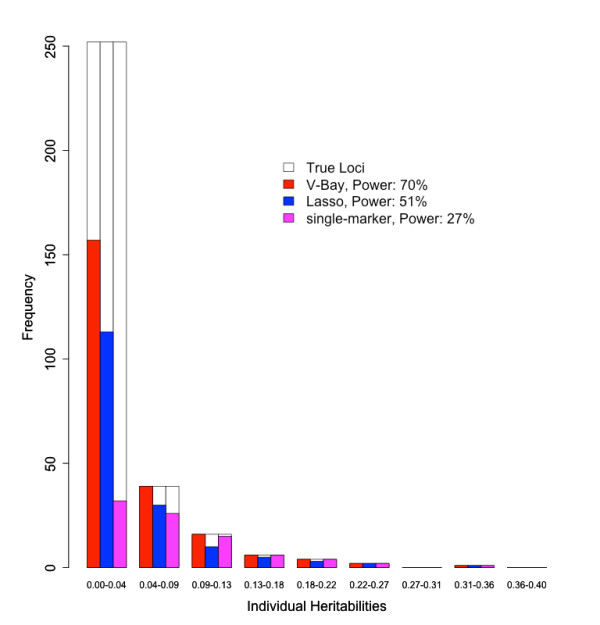
**Histograms of loci identified by V-Bay, the lasso, and single-marker analysis as a function of individual heritability**. The false-discovery rate is controlled to 0.0%. These graphs summarize the results of ten replicate simulated data-sets with 100,000 markers, 32 loci with associations, a sample size of 1000, and a total phenotype heritability of 0.9. The power for each method at 0.0% false-discovery rate is shown in the legend.

Figure 4 presents the power of V-Bay, the lasso, and single-marker analysis for simulations with one-hundred thousand markers, 32 loci, and 1000 samples, when the false-discovery rate is controlled to 0%. V-Bay, the lasso, and single-marker analysis can all correctly detect a high proportion of loci in the upper tail of the distribution, where the individual heritabilities of associations are high. However, there is variability in the number of smaller heritability loci detected, with multiple locus methods performing better. The reason for this result is when multiple locus methods correctly identify loci with larger individual heritabilities, they directly account for the effect of these loci in the statistical model. This shrinks the estimate of the error term, which increases the power to detect loci with even weaker associations. For these simulations, V-Bay outperforms not only single-marker analysis, but also the lasso. We found V-Bay performed better than the lasso (and single-marker analysis) for additional architectures and sample sizes, when controlling the false discovery rate to 5.0% (Table 3).

**Table 3:** Power comparison for V-Bay, the lasso, and single-marker GWA analysis from simulated data with 100,000 markers.

sample	loci	V-Bay	the lasso	single-marker
200	4	90.0%	87.5%	47.5%
200	32	14.1%	4.69%	7.19%
1000	4	97.5%	77.5%	60.0%
1000	32	80.6%	65.0%	33.1%

Phenotypes were simulated with a fixed total heritability of 0.9. The false discovery rate was controlled to 5% for all three analyses.

### Genome-wide association analysis of HapMap gene expression

To investigate the empirical properties of V-Bay, we performed a GWA analysis on gene expression levels measured in eternal lymphoblastoid cell lines, generated from the 210 unrelated individuals of Phase II of the International HapMap project [31]. Individuals in this sample were genotyped for upwards of 3.1 million SNPs and were derived from four populations: Caucasian with European origin (CEU), Chinese from Beijing (CHB), unrelated Japanese from Tokyo (JPT), and Yoruba individuals from Ibadan, Nigeria (YRI) [32]. In the original GWA analysis of these data, Stranger et al. used a single-marker testing approach, considering each population independently, and limiting the analysis to SNPs in the *cis*-regions of each gene to control the level of multiple test correction [31].

Using a version of V-Bay that accounts for population structure and missing genotype data, we analyzed the pooled data from these populations. We did not limit the analysis to *cis*-regions, although we did limit our analyses to SNPs with MAF > .10, leaving 1.03 million markers genome-wide. To minimize computational cost, we also limited our analysis to the 100 expression probes Stranger et al. found to have the most significant associations, and an additional 20 probes with the largest residual variance, after correcting for population structure. For comparison, we also applied a single-marker analysis to these pooled data, for the 120 expression probes, incorporating a covariate to account for population structure.

**Figure 5:**
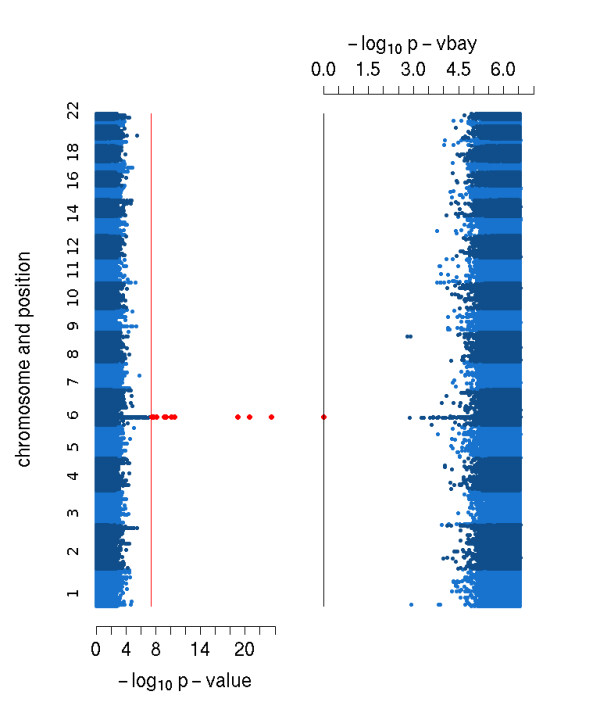
**Manhattan plots of the results of a single-marker (left) and V-Bay GWA analysis (right) of the gene expression product HLA-DRB1 for individuals in HapMap**. Each dot reflects the -log_10 _p-value resulting from the single-marker analysis (left) and the -log10 p-vbay output of V-Bay (right), where non-significant associations are represented as blue dots (alternating shades are used to distinguish chromosomes). The markers above the red line for the single-marker analysis are significant when using a Bonferroni correction. The marker in red for the V-Bay analysis (in the black line) is significant at an equivalently conservative false positive control. Note that the lasso was also able to identify this association. We did not incorporate the SNPs on the X and Y chromosomes in our analyses.

On average, V-Bay was able to complete the GWA of each of these expression phenotypes in 1.5 hours using a dual-quad core Xeon 2.8 Ghz (16 Gb of memory). In 90% of cases, where our single-marker analysis reproduced the most significant *cis*-associations reported by Stranger et al., V-Bay also identified the association. In addition, a total of 72 out of the 100 previously reported *cis*-associations [31] were identified with V-Bay (Additional file 1, **Table S1**). A typical result from these analyses is presented in Figure 5. These Manhattan plots are for the HLA-DRB1 expression probe, which was not reported by Stranger et al. as having a strong *cis*-association. For this probe, V-Bay, the lasso, and our multiple population single-marker analysis indicated a strong *cis*-association. Since this association was also found with single-marker analysis, identification was not due to V-Bay but to the analysis of the pooled data from different populations (as opposed to testing within populations as in Stranger et al. [31]). Still, the increased sensitivity of V-Bay was suggested in this case by *trans*-associations identified by individual runs of V-Bay, which were not identified by the single-marker analysis or the lasso. However, we imposed the restrictive criteria that an association identified by V-Bay would only be considered significant if it was robust to missing data resampling and marker reordering runs. Using this conservative strategy, none of the putative *trans*-associations were robust enough to report. With an increased sample size, we believe that these *trans*-associations could be confidently assigned as true hits.

## Conclusions

V-Bay addresses computational efficiency and performance concerns associated with many multiple locus GWA algorithms. While V-Bay currently utilizes a hierarchical partitioning model, the same approach could be used to implement scalable algorithms for a wide range of models. For example, different shrinkage or penalization models such as the lasso [11,14], ridge regression [24], or a normal exponential gamma distribution penalty [4] are easily implemented by removing the partitioning and substituting the appropriate prior distribution. Further, the variational Bayes method used for computation does not require specific closed form integrals arising from hyperparameter distributions, which characterize many of the proposed algorithms for full penalized-likelihood or Bayesian GWA analysis [4,11,24]. There is therefore the potential for developing an entire class of scalable multiple locus algorithms for GWA analysis that could be tuned for different genetic and experimental conditions within the V-Bay framework.

## Methods

### V-Bay Algorithm

The V-Bay algorithm consists of two components, a hierarchical regression model with marker class partitioning and a variational Bayes computational algorithm. The hierarchical regression is adapted directly from Zhang et al. [13] with minor alterations. The first level of the hierarchical regression model for a sample of *n *individuals with *m *markers is a standard multiple regression model:(1)

where *y*_*i *_is the phenotype of the *i*^*th *^individual, *μ *is the sample mean, *x*_*ij *_is the genotype of the *j*^*th *^marker of the i^*th *^individual, *β*_*j *_is the effect of the *j*^*th *^marker, and *e*_*i *_~ N . While we limit the current presentation of the model to continuous traits with normal error, more complex error structures and extensions to discrete traits is straightforward. Because (1) is a linear model, it can be easily expanded to test for dominance or epistasis using a standard mapping approach. In addition, confounding factors such as population structure can be accounted for by the addition of covariates. The effects of these additional covariates can be modeled within the hierarchical regression framework or can be treated simply as nuisance parameters and given uninformative priors. We used an uninformative prior  for the error parameter, , and a constant (improper) prior for the mean parameter *μ*.

The second level of the hierarchical model consists of a partitioning of markers into positive, negative, and zero effect classes and the prior control over the distributions of these classes. The partitioning is accomplished by modeling each of the regression coefficients using mixture prior distributions:(2)

where  is an indicator function for *β*_*j *_with a value of zero, and N_+ _and N_- _are positive and negative truncated distributions [13]. The priors on the population distribution of positive and negative effect probability hyperparameters ( and ) are:(3)

In our analyses we chose an uninformative Dirichlet prior by setting the parameters *θ*_*β*_, *ϕ*_*β*_, *ψ*_*β *_all to one. The hyperparameters  and  reflect the partitioning aspect of the model. Within the positive and negative partitions, the population variance parameters ( and ) have  priors. This choice of prior for the regression coefficients in the positive and negative effect classes increases the robustness to outliers. Assuming the number of markers in the GWA data set, *m*, is greater than the sample size, *n*, we truncate the Dirichlet distribution such that , where the truncation puts a lower bound on the harshness of shrinkage [8]. We found this truncation very important when considering data sets with large numbers of markers. Without truncation, the evidence in the data is too weak to enforce harsh enough shrinkage for desirable model selection.

The variational Bayes component of V-Bay is constructed by approximating the joint posterior density of the hierarchical model:(4)

in terms of a factorized form:(5)

and then minimizing the KL-divergence between the factorized and full form. Equation (5) is a natural factorization for the V-Bay hierarchical model since most of the priors are conjugate. The posterior factorized distributions all have closed form expressions and each parameter is completely characterized by an expected sufficient statistic [27] (Additional file 1, **Methods**). The algorithm is therefore equivalent to updating these expected sufficient statistics.

Minimizing the KL-divergence between each marginal distribution (e.g. *q*(*β*_*j*_)) and the full joint distribution is performed by considering the expectation of the full log joint distribution with respect to each parameter. For a generic parameter *θ*, the expectation step is equivalent to setting:(6)

with C some normalizing constant, and E_-*θ *_indicating expectation of the log of equation (4) with respect to every other parameter's factorized distribution, except *q *(*θ*). This defines a system of equations which can be iterated through until convergence [23,27]. With the factorized form, it is a simple matter to demonstrate the time complexity of V-Bay is (*nm*) per iteration (Additional file 1, **Methods**).

### V-Bay Convergence

The factorization of equation (4) is used to define a function ℒ(*θ*) which lower bounds the log posterior probability of the data (i.e. the probability of the observed data after integrating out all parameters in the model). The lower bound ℒ(*θ*) is defined as the expectation of the log of equation (4) with respect to every factorized distribution plus the entropy of each factorized distribution. In the full form, the convergence of V-Bay to a local maximum of the lower bound ℒ(*θ*) is guaranteed because of the convexity of ℒ(*θ*) with respect to each parameter's approximate posterior distribution [33]. In the described implementation we used an approximation for some higher order expectation terms that we found increased computational efficiency (Additional file 1, **Methods**).

Given that global convergence to a single stationary point is not guaranteed [26], the standard practice is to use multiple parameter initializations. We found that with random initializations of expectations of *β*_*j*_, V-Bay finds local modes that correspond to over-fit (under-determined) models, while with initializations of only a few non-zero expectations of *β*_*j*_'s, V-Bay tends to update these values close to zero before converging. We therefore use the approach of setting all expectations of *β*_*j *_parameters equal to zero as a starting point for all runs of V-Bay, an approach that has precedent in simultaneous marker analysis [4]. This also corresponds to appropriate starting estimates given our prior assumption that not too many markers are associated with a phenotype.

We have found that the order in which the parameters are updated can affect local convergence, particularly when there is missing genetic data. In general, the different association models we found using different orderings were not widely different from one another, often differing in whether they included one or two specific associations. For cases where we found ordering did make a difference, we ran V-Bay with multiple random orderings and used the conservative criteria of considering only associations found to be significant in at least 80% of the cases to be true positives for all simulations and data analyses compared to single-marker analysis. The cutoff of 80% corresponds directly to a false discovery rate of 0%. We also considered a less stringent cutoff and an observed false discovery rate of 5% in the comparison to the lasso.

### V-Bay Software

An implementation of V-Bay is available at http://mezeylab.cb.bscb.cornell.edu/Software.aspx. The software has basic control parameters available to the user and only requires tab delimited genotype and phenotype files as input. The algorithm itself consists of the following steps: 1) randomize marker ordering, 2) initialize the expected sufficient statistics and expectations of parameters, 3) update the expected sufficient statistics for a particular parameter, given the expectations of all the other parameters, 4) update the expectations of a particular parameter given the expectations of all the other parameters, 5) repeat steps 3 and 4 for all the parameters in the model, 6) check convergence based on the current estimate of the lower bound, ℒ(*θ*). Further functional details are presented in Additional file 1, Tables S3-S9. The main output from the algorithm is the -log_10 _of p-vbay = *p*_*j*+ _+ *p*_*j*- _statistic for each marker, which can be used to assess significance of a marker association.

### The Lasso

Originally proposed by Tibshirani [34], recently applied to GWA data by Wu et al. [14] and modified by Hoggart et al. [4], the lasso is a form of hierarchical regression that imposes a double exponential prior on the coefficients of each marker. Although expressed in a Bayesian context, maximum *a posteriori *(MAP) estimates are obtained by maximizing the following penalized log-likelihood:(7)

where ℓ(*β*|*Y*) is the log-likelihood for the relevant generalized linear model. By penalizing the magnitude of each *β*_*j *_coefficient, MAP estimates shrink the coefficient values compared to the estimates under the unpenalized model. This shrinkage causes most coefficients to be exactly zero, so that only very few markers are selected to be nonzero for a single value of *λ*. This penalty produces a convex log-likelihood surface with a single maximum even for underdetermined systems (i.e. when there are more markers than samples). Therefore, the lasso can jointly consider all markers in a single model and simultaneously account for variance in the response caused by multiple markers. The lasso model is fit for multiple values of *λ *and a single subset of coefficients is selected to be nonzero by 10-fold cross-validation. Confidence scores are obtained for each selected marker by comparing an unpenalized model with all selected markers to a model that omits each marker in turn. An F-test is performed for each marker, but note that these confidence scores cannot be interpreted as typical p-values since they are obtained from a two step procedure. Algorithmic details for fitting the LASSO model for the linear-Gaussian case are provided by [35,36].

### Simulation Study

GWA data were simulated under the set of conditions listed in Table 1. The genomic marker data were generated using MaCS [29], a scalable approximate coalescent simulator, using the default approximation tree width. For the comparison to single-marker analysis, three basic types of genotype data sets were simulated. For the first and second type, 0.5 Gb of DNA was simulated from a single diploid population with *N*_*e *_= 10000, the population scaled mutation rate 4*N*_*e*_*μ *= *θ *= 0.001, and the genome-wide population scaled recombination rate 4*N*_*e*_*κ *= *ρ *= .00045, values taken from Voight et al. [37]. Samples of 200 and 1000 were sampled screening the minor allele frequency (MAF) to be 0.10, leaving more than one-million markers for analysis. For the third type, 200 diploid samples of 0.5 Gb were simulated from a simple four population migration model. The approximation , as observed in the overall Phase I HapMap analysis [38], was used to determine the population per generation migration rate for a simple symmetric island migration model, with populations of equal size. After screening MAF to be > 0.10, this left over 660 thousand markers for analysis. The final data included the addition of 2% missing data.

Given the simulated genotypic data, phenotypic data were produced with a simple additive linear model as shown in equation (1). The genotypes were represented in the linear model with a consistent dummy variable encoding of {0, 1, 2} across loci. The additive effects were drawn independently from a Γ(2, 1) distribution or from a model with fixed effects. The locations for loci were randomly sampled throughout the genome. For each genomic data set, 4, 8, or 32 loci with phenotype associations were simulated. The total heritability of the phenotype was fixed at either 0.5 or 0.9. The MAF is computed for each sampled locus in the genetic model since each locus is chosen from the SNPs generated by MaCS. By combining the MAF with the effects sampled for each locus in the genetic model, it is possible to determine the proportion of observed variation contributed by each locus. This individual heritability for each locus is defined as follows:(8)

where *f*_*j *_is the MAF of locus *j*, *β*_*j *_is the additive effect of the locus *j*, and  is the total phenotypic variance of the trait.

GWA analysis of the simulated data were performed using both V-Bay and a linear regression single-marker analysis. When population structure was incorporated, the linear model (1) becomes a fixed effect ANOVA model, for both V-Bay and the single-marker analysis. The population means in V-Bay were treated as having normal priors centered on zero with a very large variance (*τ *= 1000), and were updated in a similar fashion as the other parameters in the V-Bay algorithm. The V-Bay algorithm was run until the tolerance for the likelihood portion of the lower bound ℒ(*θ*) was < 10^-9^. For the simulations with missing data, the minor allele frequency across loci (*f*_*j *_∀*j*) was estimated given the observed genotype data, and then the missing data points were sampled from a *Bin*(*n *= 2, *f*_*j*_), i.e. assuming Hardy-Weinberg equilibrium, for both V-Bay and single-marker analysis. We did random re-sampling of missing data to test the robustness of the output of V-Bay and the single-marker analysis (Additional file 1, **Methods**).

The false positive and true positive rates were calculated for each set of replicate simulations. Care was taken to account for the effect of linkage disequilibrium on the test statistics, for both V-Bay and single-marker analysis. A simple window was computed around each marker to determine when the *r*^2 ^decayed to 0.4. The cutoff of 0.4 was used to be as generous to single-marker analysis as possible. Any marker in this window was considered a true positive. In the case where multiple recombination events occurred recently between different ancestral lineages, multiple blocks of markers in linkage disequilibrium were generated, that were separated by markers in low linkage disequilibrium. In these cases, a conservative rule for evaluating a true positive was implemented. If a marker had a p-vbay> 0.99, or -log_10 _p-value for the single-marker analysis in excess of the Bonferroni correction, and the *r*^2 ^between the significant genetic marker and the true location was greater than 0.4, then the marker was considered a true positive.

For the comparison between V-Bay, the lasso, and single marker analyses, one-hundred thousand markers and samples sizes of 200 or 1000 for a single population were simulated (the reduced number of markers for these simulations was used to conserve CPU cycles). The genetic architectures were simulated as with the larger scale simulations, but with only 4 or 32 loci being sampled randomly from the one-hundred thousand markers, and effects sampled from a Γ(2, 1) distributions for 10 replicated data sets. Eight random reorderings of the markers were used with the V-Bay analysis, and the false discovery rate for V-Bay was controlled based on the consensus of associations found across reorderings with p-vbay> 0.99 (e.g. a false discovery rate of 5% corresponded to an association being found in at least 3 out of the 8 reorderings). The false discovery rate for the lasso (using F-statistics) and single-marker analysis were controlled based on the p-values computed for each method respectively.

### Data Analysis

We performed a GWA analysis for gene expression levels measured in the eternal lymphoblastoid cell lines that were generated for the 210 unrelated individuals of Phase II of the International HapMap project [31]. This sample included 60 individuals sampled from Utah of European descent (CEU), 45 individuals sampled from Han Chinese population (CHB), 45 individuals sampled from Japanese population (JPT), and 60 individuals sampled from the Yoruban population in Africa (YRI). Expression data for these lines were available for 47,000 probes for (~17,000 genes) assayed with the Illumina bead array. For our analyses, we screened for MAF > 0.10 in all populations which left 1.03 * 10^6 ^SNPs on chromosomes 1 to 22. The X and Y chromosomes were not analyzed by Stranger et al. and we ignored these chromosomes in our analyses as well. Stranger et al. [31] reported 879 gene expression probes with highly significant *cis*-eQTL associations, found by testing within populations, where every SNP in a 2 Mb window around each gene was analyzed. We performed a GWA analysis, with both V-Bay and a single-marker regression, for their top 100 most significant expression probes. We combined genotypic data across populations, where we accounted for the effect of population structure in each case by including appropriate covariates. We also tested the top 20 probes, not in their association list that had the largest residual variance after correcting for population structure. Only 120 expression probes were analyzed to conserve CPU cycles; all 879 could easily be analyzed in a future study. The total missing data for this SNP set was 1.78%. We accounted for missing data using the same approach as with our simulated data analysis.

## Availability and Requirements

Both binaries and source code for the V-Bay software are available at the following URL: http://mezeylab.cb.bscb.cornell.edu/Software.aspx. The source code is released under the GNU General Public License http://www.gnu.org/licenses/. The binary was compiled for 32-bit architecture on Ubuntu 8.04 http://www.ubuntu.com/ using the compiler gcc http://gcc.gnu.org/ and the GNU scientific library http://www.gnu.org/software/gsl/. To recompile from source both gcc and GSL are required. Documentation describing how to use V-Bay as well as example data sets are also available at http://mezeylab.cb.bscb.cornell.edu/Software.aspx.

## Abbreviations

V-Bay: Variational Bayes algorithm for genome-wide association analysis; GWA(S): Genome-Wide Association (Study); MAF: Minor Allele Frequency; eQTL: expression-Quantitative Trait Loci; ANOVA: Analysis of Variance; EM: Expectation-Maximization algorithm; SNP: Single Nucleotide Polymorphism; ROC curve: Receiver Operating Characteristic curve; Lasso: Least Absolute Shrinkage and Selection Operator.

## Authors' contributions

BAL developed and implemented the V-Bay algorithm, performed the simulation and data analyses, and wrote the software. BAL and JGM designed the simulation and data analyses. GH implemented the lasso algorithm, and ran the lasso algorithm for the simulation and data analyses. BAL and JGM wrote the manuscript. All authors read and approved the final version of this manuscript.

## Supplementary Material

Additional file 1 Portable Document File (PDF) containing additional results and methods that are referred to in the text. Click here for file
