# Supplementary material for: A variational Bayes algorithm for fast and accurate multiple locus genome-wide association analysis
Source: BMC Bioinformatics. 2010 Jan 27;11:58. doi: 10.1186/1471-2105-11-58 (PMC2824680; doi:10.1186/1471-2105-11-58)
Supplement: Additional file 1 — Portable Document File (PDF) containing additional results and methods that are referred to in the text. [file 1471-2105-11-58-S1.PDF]

Supplementary Results and Methods

Benjamin A Logsdon, Gabriel E Hoffman, Jason G Mezey

Supplementary Results

Data analysis. We chose a subset of eleven gene expression phenotypes from the Stranger et al. study [1] that contained putative *trans*-associations from a single run of the algorithm (for a single sampling of missing data). We did both completely random reordering of the markers as well as resampling of the missing data. In all cases the putative *trans*-associations were not robust under the random reorderings and resampling, hence we only reported the *cis*-associations (identified by both V-Bay and our single-marker reanalysis). We report an additional 61 *cis*-associations (along with these 11) that were identified with both V-Bay and single-marker analysis under a single run of V-Bay in **Supplementary Tables 1a and 1b**.

Supplementary Table 1a HapMap Phase II gene expression reanalysis results.

GENE	SNP ID	Position	Chromosome
FLJ10781	rs12978825	51666146	19
UGT2B17	rs3100645	69525783	4
UGT2B11	rs2708697	69031629	4
GSTM1	rs366631	110052995	1
C14orf52	rs10132742	64456675	14
KIAA1463	rs3742062	49415099	12
UBA2	rs2314664	18552942	19
GSTT1	rs407257	22676550	22
Hs.396207	rs3014241	45860466	1
UGT2B7	rs2708697	69031629	4
FLJ46603	rs1014390	72224481	17
LOC284293	rs6567407	5978905	18
LOC51240	rs1233276	190393027	2
USMG5	rs11191688	105182560	10
MRPL43	rs10786612	102643755	10
MGC2752	rs7249714	63749895	19
PKHD1L1	rs1026437	110562125	8
PHACS	rs2074040	44049899	11
LOC283970	rs6499292	68661559	16
IRF5	rs10229001	128386633	7
hmm1412	rs747172	70242799	11
NUDT2	rs4310287	34364979	9
FLJ21616	rs1487969	28941580	8
PTER	rs4748302	16595868	10
Hs.400876	rs752775	36488195	20
AXIN1	rs214249	288688	16
TINP1	rs6883061	74128556	5
LOC284184	rs11150780	76878755	17
FLJ21347	rs6504675	45989356	17
RPL37A	rs284565	217067051	2
LOC375097	rs752775	36488195	20
C21orf107	rs2836934	39486755	21
LCMT1	rs7188975	25044950	16
MRPL43	rs10786612	102643755	10
hmm8232	rs3863641	46615803	1
CCNDBP1	rs2412752	41127265	15

Supplementary Table 1b HapMap Phase II gene expression reanalysis results.

GENE	SNP ID	Position	Chromosome
PLOR2E	rs3787016	1041803	19
LOC378075	rs2419490	64907258	7
LOC400642	rs9948693	5237432	18
KIAA1913	rs4897398	130649264	6
XRRA1	rs2298746	74231482	11
EIF2S1	rs1078194	66777549	14
SYNGR1	rs909685	38077617	22
PEX6	rs2274514	43042478	6
FLJ90036	rs6814287	112323	4
QRSL1	rs6568448	107200445	6
LOC339804	rs1177303	61241859	2
Hs.453941	rs880034	119702442	8
CDK5RAP2	rs2297454	122211576	9
NUDT2	rs7039222	34322740	9
VPS13A	rs1054368	78981613	9
LOC339229	rs3830068	77233294	17
PPA2	rs13108489	106512574	4
KIAA1712	rs4695916	175437965	4
Hs.519979	rs3862293	2992845	6
MGC12458	rs1979568	243259095	1
MGC22773	rs792310	74438081	1
STK25	rs2240482	242053684	2
HSRTSBETA	rs2305995	683977	18
UGT2B10	rs3100651	69495658	4
LOC400933	rs10854876	48396574	22
dJ383J4.3	rs1951626	172158724	1
LOC197322	rs12931350	87735816	16
Hs.6637	rs2293577	47393768	11
FLJ32112	rs12046885	54329878	1
Hs.379903	rs9891938	15855797	17
Hs.26039	rs2279327	10709785	5
WBSCR27	rs4304218	72890916	7
ST7L	rs7415820	112970972	1
HLA-DQA2	rs9275312	32773706	6
hmm26268	rs11118858	220019581	1
OAS1	rs7134391	11851074	12

Supplementary Methods

V-Bay algorithm steps. The algorithm proceeds as follows: 1) Initialize all expected sufficient statistics and expectations for β_j parameters and the expectation of μ to 0. Initialize expectations for $p_{\beta+}$, $p_{\beta-}$ parameters to $\frac{1}{3}$. Initialize expectations of variance parameters σ_e^2 and hyperparameters $\sigma_{\beta+}^2$,$\sigma_{\beta-}^2$ to 1. 2) Compute the likelihood portion of the lower bound, $\mathcal{L}(\theta)$. The likelihood component of $\mathcal{L}(\theta)$ was a very practical convergence diagnostic in terms of computational efficiency. 3) Update the expected sufficient statistics and expectation of the μ parameter. 4) Update the expected sufficient statistics and expectations for each β_j parameter. 5) Update the expected sufficient statistics and expectations for the error term σ_e^2. 6) Update the expected sufficient statistics and expectations for the variance hyperparameters $\sigma_{\beta+}^2$, $\sigma_{\beta-}^2$. 7) Update the expected sufficient statistics and expectations for the probability of effect hyperparameters $p_{\beta+}$, $p_{\beta-}$. 8) Repeat steps 2-7) until the difference in lower bound between updates is less than 10^{-9}. 9) Return the sufficient statistics, specifically the p_{j+} and p_{j-} parameters (see **Supplementary Table 3**).

Expected sufficient statistics and expectations of parameters. The population mean μ has a normal approximate factorized posterior, and is therefore characterized by a mean μ_μ and variance σ_μ^2 illustrated in **Supplementary Table 2**. The expectation $\mathrm{E}[\mu]$ is just the mean statistic, μ_μ.

The factorized approximate posterior density for each β_j parameter is a mixture distribution characterized by six sufficient statistics, a (posterior) positive effect mean μ_{j+}, a (posterior) negative effect mean μ_{j-}, a (posterior) positive effect variance σ_{j+}^2, a (posterior) negative effect variance σ_{j+}^2, a (posterior) probability of positive effect p_{j+}, and a (poste-

rior) probability of negative effect p_{j-}. **Supplementary Table 3** shows the expectations of these sufficient statistics in terms of the expectations of other parameters in the model. The functions $\phi(x)$ and $\Phi(x)$ are the standard Normal probability density and cumulative density functions respectively. Once the expected sufficient statistics for β_j are computed, the necessary expectations of β_j can be computed, $\mathrm{E}[\beta_j]$ and $\mathrm{E}\left[\beta_j^2\right]$ as shown in **Supplementary Table 4**. Note that $\mathrm{E}[p_{\beta+}]$ and $\mathrm{E}[p_{\beta-}]$ are used instead of $\exp(\mathrm{E}[\log\{p_{\beta+}\}])$ and $\exp(\mathrm{E}[\log\{p_{\beta+}\}])$ respectively for computational convenience, which we found did not affect the performance of the algorithm significantly (results not shown).

The approximate factorized posterior for the inverse of the error variance, σ_e^{-2} is characterized by a Gamma distribution, hence has shape and scale sufficient statistics, $\nu_e = \frac{n}{2}$ and ρ_e shown in **Supplementary Table 5**. Some of the higher order terms are dropped here from ρ_e (specifically the $\mathrm{E}\left[\beta_j^2\right]$ terms) for ease of computation. Again, we found that this did not significantly affect the performance of the algorithm (results not shown). The expectation for σ_e^{-2} is therefore $\mathrm{E}\left[\sigma_e^{-2}\right] = \nu_e \rho_e$.

Next we turn to the expected sufficient statistics for the positive and negative effect class variance hyperparameters, $\left(\sigma_{\beta+}^{-2}, \sigma_{\beta-}^{-2}\right)$. Since the priors for these are χ_1^2, the approximate posterior distribution for each parameter is a Gamma distribution, characterized by two sufficient statistics, a shape statistic ν_+ or ν_-, and a scale statistic ρ_+ or ρ_- as shown in **Supplementary Table 6**. Then the expectations, $\left(\mathrm{E}\left[\sigma_{\beta+}^{-2}\right], \mathrm{E}\left[\sigma_{\beta-}^{-2}\right]\right)$, can be computed as shown in **Supplementary Table 7**.

In addition, the expected sufficient statistics for the probability of membership in the positive, negative, and zero effect classes $(\Theta_\beta, \phi_\beta, \Psi_\beta)$ are illustrated in **Supplementary**

Table 8. A uniform prior was assumed for this Dirichlet distribution. To compute the expectations with a truncated Dirichlet prior we used the property that any pairwise marginal distribution of the Dirichlet distribution is a Beta distribution. We used the marginal distribution with the positive and negative effect classes pooled since this is how the truncation was defined: $p_{\beta+} + p_{\beta-} \leq \frac{\sqrt{n}}{m}$. With the Beta distribution we used the GNU Scientific Library [2] to access the incomplete Beta function to compute the necessary expectations, $(\mathrm{E}\left[p_{\beta+} + p_{\beta-}\right], 1 - \mathrm{E}\left[p_{\beta+} + p_{\beta-}\right])$ for a truncated Beta distribution. Then, we used the relative proportion of evidence in the positive and negative effect class to evaluate the expectation for the classes (e.g. $\mathrm{E}\left[p_{\beta+}\right] = \mathrm{E}\left[p_{\beta+} + p_{\beta-}\right] \frac{\Theta_\beta}{\Theta_\beta + \Phi_\beta}$).

Population structure. The population structure version of the algorithm has additional population mean parameters α_l for k populations incorporated into the linear model in equation (1) in the main text. The same factorization as in equation (5) in the main text is assumed over the posterior distribution of the α_l parameters. A normal prior with large variance is applied to each α_l parameter, leading to update equations similar to those in **Supplementary Table 3**, except the approximate posterior density is no longer a mixture density but just a Normal distribution characterized by mean and variance sufficient statistics (not shown, available on request).

Additional algorithmic details. For data sets with marker number $\geq 100,000$, the numerical library we used to evaluate the truncated expected sufficient statistics for the $\mathrm{E}\left[p_\beta\right]$ terms did not have high enough numerical precision to prevent underflow for large values of the sufficient statistics Θ_β and Φ_β. We therefore used a harsh update $\frac{\sqrt{n}}{100m}$ for each expectation $\mathrm{E}\left[p_{\beta+}\right]$ and $\mathrm{E}\left[p_{\beta-}\right]$ for the initial iterations where the sufficient statistics were large. In general, once the sufficient statistics shrink sufficiently (so as to be on the

order of $\frac{\sqrt{n}}{m}$), then the truncated expectations $\mathrm{E}\left[p_\beta\right]$ can be computed exactly. We empirically found this to be the best trade-off between converging to a suboptimal over-fit model with hundreds of significant markers for too weak of an approximate update and too harsh of an update where we lose significant power (results not shown). For smaller marker numbers, $m \leq 100,000$, this was not a problem.

$\mathcal{O}\left(nm\right)$ **complexity for a single update.** As demonstrated by the form of μ_{j+} or μ_{j-} in **Supplementary Table 3**, the most computationally intensive step in the algorithm occurs during the update of the expected sufficient statistic for the β_j parameters. This is because the residual term $\sum_{l \neq j} \mathrm{E}\left[\beta_l\right] x_{li}$ must be recomputed for each step. Most of the terms in summation in this expression stay the same for updates of different β_j parameters. Hence, we store this residual term as a vector of length n and for any particular update of the expected sufficient statistics of a new β_j parameter we add or subtract the necessary terms (e.g. subtract the term $\mathrm{E}\left[\beta_j\right] x_{ji} \forall\ i$ from this residual when updating μ_{j+} and μ_{j-} for the β_j parameter). The update of a single β_j expected sufficient statistic therefore has $\mathcal{O}\left(n\right)$ complexity. Because there are m β_j parameters the total time complexity of a single update of all β_j parameters is $\mathcal{O}\left(nm\right)$. The complexity of updating the other parameters is either linear in terms of the sample size n or marker number m (e.g. updating σ_e^2 has complexity $\mathcal{O}\left(n\right)$, and updating $\sigma_{\beta+}^2$ has complexity $\mathcal{O}\left(m\right)$.) Therefore the total time complexity of the algorithm is $\mathcal{O}\left(nm\right)$.

Supplementary Tables

Supplementary Table 2 Expected Sufficient Statistics for μ.

	Expected Statistic
μ_μ	$\frac{1}{n}\sum_{i=1}^{n}\left(y_i - \sum_{j=1}^{m}\mathrm{E}\left[\beta_j\right]x_{ji}\right)$
σ_μ^2	$\frac{\mathrm{E}\left[\sigma_e^2\right]}{n}$

Supplementary Table 3 Expected Sufficient Statistics for β_j.

	Expected Statistic
μ_{j+}	$\frac{\mathrm{E}\left[\sigma_{\beta+}^2\right]\sum_{i=1}^{n}x_{ji}\left(y_i-\mathrm{E}[\mu]-\sum_{l\neq j}\mathrm{E}[\beta_l]x_{li}\right)}{\mathrm{E}[\sigma_e^2]+\mathrm{E}\left[\sigma_{\beta+}^2\right]\sum_{i=1}^{n}x_{ji}^2}$
μ_{j-}	$\frac{\mathrm{E}\left[\sigma_{\beta-}^2\right]\sum_{i=1}^{n}x_{ji}\left(y_i-\mathrm{E}[\mu]-\sum_{l\neq j}\mathrm{E}[\beta_l]x_{li}\right)}{\mathrm{E}[\sigma_e^2]+\mathrm{E}\left[\sigma_{\beta-}^2\right]\sum_{i=1}^{n}x_{ji}^2}$
σ_{j+}^2	$\frac{\mathrm{E}\left[\sigma_{\beta+}^2\right]\mathrm{E}\left[\sigma_e^2\right]}{\mathrm{E}[\sigma_e^2]+\mathrm{E}\left[\sigma_{\beta+}^2\right]\sum_{i=1}^{n}x_{ji}^2}$
σ_{j-}^2	$\frac{\mathrm{E}\left[\sigma_{\beta-}^2\right]\mathrm{E}\left[\sigma_e^2\right]}{\mathrm{E}[\sigma_e^2]+\mathrm{E}\left[\sigma_{\beta-}^2\right]\sum_{i=1}^{n}x_{ji}^2}$
p_{j+}	$\frac{2\mathrm{E}\left[p_{\beta+}\right]\frac{\sigma_{j+}}{\sqrt{\mathrm{E}\left[\sigma_{\beta+}\right]}}\Phi\left(\frac{\mu_{j+}}{\sigma_{j+}}\right)\exp\left\{\frac{\mu_{j+}^2}{2\sigma_{j+}^2}\right\}}{1-\mathrm{E}\left[p_{\beta+}\right]-\mathrm{E}\left[p_{\beta-}\right]+2\mathrm{E}\left[p_{\beta+}\right]\frac{\sigma_{j+}}{\sqrt{\mathrm{E}\left[\sigma_{\beta+}\right]}}\Phi\left(\frac{\mu_{j+}}{\sigma_{j+}}\right)\exp\left\{\frac{\mu_{j+}^2}{2\sigma_{j+}^2}\right\}+2\mathrm{E}\left[p_{\beta-}\right]\frac{\sigma_{j-}}{\sqrt{\mathrm{E}\left[\sigma_{\beta-}\right]}}\Phi\left(-\frac{\mu_{j-}}{\sigma_{j-}}\right)\exp\left\{\frac{\mu_{j-}^2}{2\sigma_{j-}^2}\right\}}$
p_{j-}	$\frac{2\mathrm{E}\left[p_{\beta-}\right]\frac{\sigma_{j-}}{\sqrt{\mathrm{E}\left[\sigma_{\beta-}\right]}}\Phi\left(-\frac{\mu_{j-}}{\sigma_{j-}}\right)\exp\left\{\frac{\mu_{j-}^2}{2\sigma_{j-}^2}\right\}}{1-\mathrm{E}\left[p_{\beta+}\right]-\mathrm{E}\left[p_{\beta-}\right]+2\mathrm{E}\left[p_{\beta+}\right]\frac{\sigma_{j+}}{\sqrt{\mathrm{E}\left[\sigma_{\beta+}\right]}}\Phi\left(\frac{\mu_{j+}}{\sigma_{j+}}\right)\exp\left\{\frac{\mu_{j+}^2}{2\sigma_{j+}^2}\right\}+2\mathrm{E}\left[p_{\beta-}\right]\frac{\sigma_{j-}}{\sqrt{\mathrm{E}\left[\sigma_{\beta-}\right]}}\Phi\left(-\frac{\mu_{j-}}{\sigma_{j-}}\right)\exp\left\{\frac{\mu_{j-}^2}{2\sigma_{j-}^2}\right\}}$

Supplementary Table 4 Expectations of β_j.

	Expectations
$\mathrm{E}\left[\beta_{j+}\right]$	$\mu_{j+} + \frac{\sigma_{j+}\phi\left(-\frac{\mu_{j+}}{\sigma_{j+}}\right)}{1-\Phi\left(-\frac{\mu_{j+}}{\sigma_{j+}}\right)}$
$\mathrm{E}\left[\beta_{j-}\right]$	$\mu_{j-} - \frac{\sigma_{j-}\phi\left(-\frac{\mu_{j-}}{\sigma_{j-}}\right)}{\Phi\left(-\frac{\mu_{j-}}{\sigma_{j-}}\right)}$
$\mathrm{E}\left[\beta_{j}\right]$	$p_{j+}\mathrm{E}\left[\beta_{j+}\right] + p_{j-}\mathrm{E}\left[\beta_{j-}\right]$
$\mathrm{V}\left[\beta_{j+}\right]$	$\sigma_{j+}^2\left(\frac{1-\frac{\mu_{j+}}{\sigma_{j+}}\phi\left(-\frac{\mu_{j+}}{\sigma_{j+}}\right)}{1-\Phi\left(-\frac{\mu_{j+}}{\sigma_{j+}}\right)} - \left(\frac{\phi\left(-\frac{\mu_{j+}}{\sigma_{j+}}\right)}{1-\Phi\left(-\frac{\mu_{j+}}{\sigma_{j+}}\right)}\right)^2\right)$
$\mathrm{V}\left[\beta_{j-}\right]$	$\sigma_{j-}^2\left(\frac{1+\frac{\mu_{j-}}{\sigma_{j-}}\phi\left(-\frac{\mu_{j-}}{\sigma_{j-}}\right)}{\Phi\left(\frac{\mu_{j+}}{\sigma_{j-}}\right)} - \left(\frac{\phi\left(-\frac{\mu_{j-}}{\sigma_{j-}}\right)}{\Phi\left(-\frac{\mu_{j-}}{\sigma_{j-}}\right)}\right)^2\right)$
$\mathrm{E}\left[\beta_j^2\right]$	$p_{j+}\left(\mathrm{V}\left[\beta_{j+}\right] + \mathrm{E}\left[\beta_{j+}\right]^2\right) + p_{j-}\left(\mathrm{V}\left[\beta_{j-}\right] + \mathrm{E}\left[\beta_{j-}\right]^2\right)$

Supplementary Table 5 Expected Sufficient Statistics for σ_e^{-2}.

	Expected Statistic
ρ_e	$2\left(\sum_{i=1}^{n}\left(y_i - \mathrm{E}\left[\mu\right] - \sum_{j=1}^{m}\mathrm{E}\left[\beta_j\right]x_{ji}\right)^2\right)^{-1}$

Supplementary Table 6 Expected Sufficient Statistics for $\left(\sigma_{\beta+}^{-2}, \sigma_{\beta-}^{-2}\right)$.

	Expected Statistic
ν_+	$\frac{1}{2} + \sum_{j=1}^{m} p_{j+}$
ρ_+	$\left(\frac{1}{2} + \frac{1}{2}\sum_{j=1}^{m}\mathrm{E}\left[\beta_j^2\right]p_{j+}\right)^{-1}$
ν_-	$\frac{1}{2} + \sum_{j=1}^{m} p_{j-}$
ρ_-	$\left(\frac{1}{2} + \frac{1}{2}\sum_{j=1}^{m}\mathrm{E}\left[\beta_j^2\right]p_{j-}\right)^{-1}$

Supplementary Table 7 Expectations of $\left(\sigma_{\beta+}^{-2}, \sigma_{\beta-}^{-2}\right)$.

	Expectations
$\mathrm{E}\left[\sigma_{\beta+}^{-2}\right]$	$\nu_+\rho_+$
$\mathrm{E}\left[\sigma_{\beta-}^{-2}\right]$	$\nu_-\rho_-$

Supplementary Table 8 Expected Sufficient Statistics for $(p_{\beta+}, p_{\beta-})$.

	Expected Statistic
Θ_β	$1 + \sum_{j=1}^{m} p_{j+}$
Φ_β	$1 + \sum_{j=1}^{m} p_{j-}$
Ψ_β	$1 + m - \sum_{j=1}^{m} p_{j+} - \sum_{j=1}^{m} p_{j-}$

References

[1] Stranger B, Forrest M, Dunning M, Ingle C, Beazley C, Thorne N, Redon R, Bird C, de Grassi A, Lee C, Tyler-Smith C, Carter N, Scherer S, Tavare S, Deloukas P, Hurles M, Dermitzakis E: **Relative impact of nucleotide and copy number variation on gene expression phenotypes**. *Science* 2007, **315**(5813):848–853.

[2] Galassi M, Davies J, Theiler J, Gough B, Jungman G, Booth M, Rossi F: **GNU Scientific Library Reference Manual , ISBN 0954161734**. *URL: http://www.gnu.org/software/gsl/* 2003.
